# Elucidating the influence of secondary nitrogen precursors on the performance of Fe–N–C catalysts for proton exchange membrane fuel cells

**DOI:** 10.1039/d5ya00357a

**Published:** 2026-02-20

**Authors:** Winnie Kong, Emre B. Boz, Marta C. Costa Figueiredo, Antoni Forner-Cuenca

**Affiliations:** a Electrochemical Materials and Systems, Department of Chemical Engineering and Chemistry, Eindhoven University of Technology PO Box 513 5600 MB Eindhoven Netherlands w.y.kong@tue.nl e.b.boz@tue.nl a.forner.cuenca@tue.nl; b Electrocatalytic Synthesis and Electrochemical Interfaces, Department of Chemical Engineering and Chemistry, Eindhoven University of Technology PO Box 513 5600 MB Eindhoven Netherlands m.c.costa.figueiredo@tue.nl

## Abstract

Proton exchange membrane fuel cells (PEMFCs) offer high efficiency, rapid refueling, and zero-carbon operation, but their commercialization is constrained by the cost of platinum-based oxygen reduction catalysts. Transition metal–nitrogen–carbon materials, particularly Fe–N–C, are promising platinum-free alternatives, although their activity and durability still require improvement. Ammonia is often introduced during synthesis to enhance nitrogen incorporation, but safer nitrogen sources are desirable to simplify processing and reduce associated hazards. Here, we investigate the use of urea, melamine, cyanoguanidine, and nicarbazin as nitrogen precursors during the thermal activation of ZIF-8-derived Fe–N–C catalysts, aiming to promote nitrogen incorporation and active-site formation without the use of ammonia. Structural analysis reveals that the use of urea, melamine, and cyanoguanidine during heat-treatment largely preserve the ZIF-8 morphology, while nicarbazin leads to the formation carbonaceous flakes. The high surface area of ZIF-8 (∼1600 m^2^ g^−1^) is partially retained after pyrolysis (∼1200 m^2^ g^−1^). X-ray photoelectron spectroscopy reveals enhanced nitrogen content and increased Fe–N_*x*_ species, most notably in urea-activated samples. Electrochemical testing in an acidic electrolyte confirms higher onset potentials and mass activities for urea- and melamine-activated catalysts compared to the control, with consistent trends observed in rotating disk electrode and single-cell PEMFC measurements. Despite their lower intrinsic activity compared to Pt/C, Fe–N–C catalysts exhibit enhanced ORR kinetics when secondary nitrogen precursors are used during synthesis. Despite elevated peroxide yields predicted from rotating ring disk electrode measurements, ion chromatography indicates a modest increase in ionomer degradation compared to Pt/C during fuel cell tests. Overall, nitrogen-rich molecular precursors enhance Fe–N–C activity while providing a safer and scalable pathway for nitrogen doping, advancing the development of cost-effective non-platinum catalysts for fuel cells.

## Abstract 2

Four nitrogen precursors are evaluated for iron–nitrogen–carbon catalysts in proton-exchange membrane fuel cells. Improved nitrogen incorporation and iron–nitrogen coordination enhance activity, linking synthesis to oxygen reduction performance.

## Graphical abstract

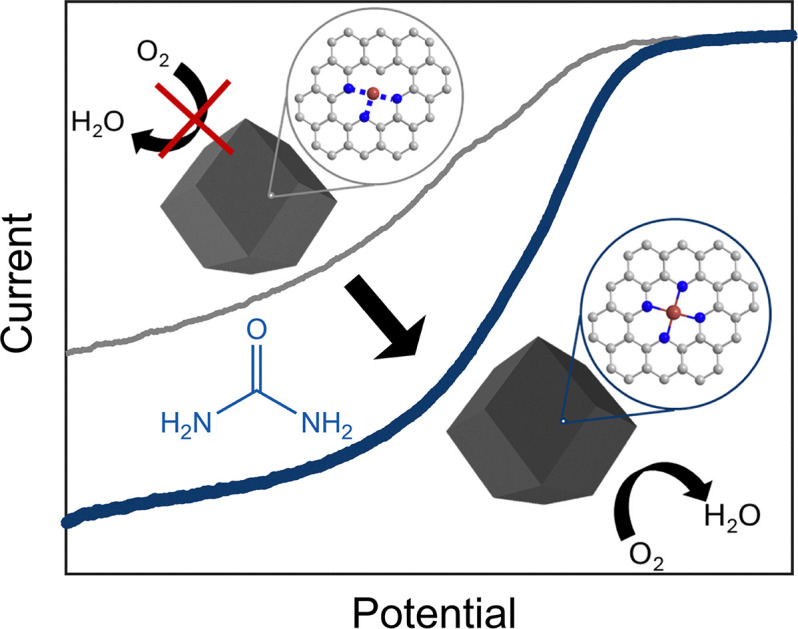

## Introduction

Growing sustainability needs for the energy infrastructure are bringing hydrogen forward as a promising energy carrier. Fuel cell technologies, particularly proton exchange membrane fuel cells (PEMFCs), have attracted significant attention as alternatives to fossil fuel-based power systems such as internal combustion engines. Compared to combustion engines, PEMFCs offer higher efficiency while maintaining a similar driving range and refueling time.^1^ Relative to batteries, they provide faster refueling and a longer driving range, but are limited by the lack of hydrogen infrastructure and the elevated cost of green hydrogen.^2,3^ Beyond their practical benefits, PEMFCs also offer environmental advantages. Hydrogen and oxygen (from air) are electrochemically converted *via* the hydrogen oxidation reaction (HOR) and the oxygen reduction reaction (ORR) to generate electricity and water as the sole product. When green hydrogen is employed, this process enables a potentially closed-loop, sustainable fuel cycle.

Like other energy technologies, large-scale implementation is constrained by cost. The catalyst accounts for 34–53% of the membrane electrode assembly (MEA) cost, depending on its loading and application,^4,5^ and platinum remains as the state-of-the-art catalyst for both the HOR and the ORR. To increase the surface area, platinum is typically dispersed as nanoparticles on conductive carbon supports, with ionomer addition enabling multiphase mass transport.^6,7^ Due to the inherently slow kinetics of the ORR, the cathode typically requires ∼4 times more platinum than the anode. As a result, significant research has focused on reducing platinum content through strategies such as intermetallic compounds and alloying,^8,9^ core–shell particles,^10^ or a redesign of the catalyst layer architecture.^11,12^ An alternative approach is the complete elimination of platinum by developing platinum group metal (PGM)-free catalysts for the ORR.^13,14^ Replacing the cathode with a platinum-free catalyst would substantially improve the cost-competitiveness of PEMFCs if the production methods are cost-effective and rely on abundant materials.^15^

Among PGM-free catalysts, transition metal–nitrogen–carbon (M–N–C) materials, where M is typically iron, cobalt, or manganese, have emerged as leading candidates.^16^ M–N–C catalysts, particularly those featuring an M–N_4_ coordination structure, exhibit promising ORR activity due to the well-defined active sites of single-atom configurations, which enable high metal utilization.^17^ The chemical state of non-PGM catalysts may locally tune the adsorption environment, improving the balance of intermediate binding energies and thereby promoting the preferred four-electron reduction to water.^18^ In acidic media, oxygen reduction can proceed *via* two associative pathways: (i) a two-electron pathway with one intermediate (*OOH) and hydrogen peroxide as the product or (ii) the full reduction to water through a four-electron pathway which involves three intermediates (*OOH, *O, and *OH).^19^ Alternatively, the dissociative pathway involves only two intermediates (*O and *OH) with water as the product, but this pathway is unattainable for single-atom sites.^18^ Consequently, non-PGM catalysts must proceed *via* the associative four-electron pathway, which inherently results in more complex kinetics and selectivity than for PGM catalysts. Therefore, establishing the correlation between the chemical state of the active site and reaction kinetics and selectivity is essential.

M–N–C catalysts are typically synthesized by pyrolyzing precursors containing nitrogen, carbon, and transition metals at high temperatures. To enhance the dispersion and accessibility of active sites, nitrogen doping and the use of metal–organic frameworks, such as zeolitic imidazolate frameworks (ZIFs), have been widely applied.^20^ Metal–organic framework-derived carbons provide high surface areas and porous structures, improving catalyst utilization and exposure of active sites.^21,22^ Such catalysts have demonstrated substantial ORR activity and initial fuel cell performance approaching Pt/C under H_2_–O_2_ conditions.^23,24^ However, their performance and stability under air operation remain insufficient for long-term applications, particularly in heavy-duty transport.^25,26^

Ongoing research aims to enhance the intrinsic activity and durability of M–N–C catalysts by optimizing their structural and electronic properties.^26^ Nitrogen content and coordination are especially critical, as nitrogen atoms form part of the catalytic sites. Higher nitrogen levels can increase active site density, while the specific coordination environment strongly influences activity and stability.^27^ Both pyridinic^28^ and pyrrolic nitrogen^29^ have been associated with enhanced ORR performance. Despite extensive work in this area, the exact roles of the nitrogen species in ORR activity and stability remain under debate.^30–32^ For example, previous research has shown that pyrrolic nitrogen modulates the adsorption of the *OOH intermediates and provides a pathway to the two-electron pathway.^33^ Additionally, protonation of the pyrrolic Fe–N_4_ sites may lead to iron leaching, while protonation of pyridinic Fe–N_4_ sites happens at the iron center, contributing to a higher barrier of iron leaching and thus higher stability.^16,34^ Regardless of the coordination environment, increased nitrogen levels enhance the density of potential active sites, which is generally beneficial for ORR performance.

Previous studies on Fe–N–C catalysts have primarily focused on nitrogen-rich polymeric precursors (*e.g.*, polyaniline and polypyrrole),^35^ added isolated small molecules such as cyanamide as precursors^36–38^ or ammonia-based post-treatments.^39–41^ Ammonia treatment during pyrolysis has been widely employed to increase nitrogen content,^39–41^ enhance microporosity,^42^ and promote pyridinic nitrogen formation.^43^ However, its use raises significant safety and scalability concerns, as ammonia is toxic and corrosive and requires specialized handling, storage, and robust ventilation systems, particularly during high-temperature processes.^44,45^ Consequently, these challenges have motivated our search for safer nitrogen precursors, such as urea and amine-functionalized molecules, which can decompose into ammonia *in situ* during pyrolysis, thereby avoiding the risk of direct ammonia handling. Urea is a well-established nitrogen precursor, offering effective doping and facilitating the formation of catalytically active sites within the carbon matrix.^46^ Similarly, amine-containing molecules such as cyanoguanidine, melamine, and nicarbazin decompose under inert thermal treatment to release ammonia, potentially providing a controlled and safer nitrogen source.^41–44^ The present work systematically compares four chemically distinct nitrogen precursors: urea, melamine, cyanoguanidine, and nicarbazin, introduced during the final activation step of a common Fe–N–C base material. This approach enables a direct assessment of precursor effects on Fe–N_*x*_ site formation and catalyst performance under realistic fuel cell operating conditions.

**Fig. 1:**
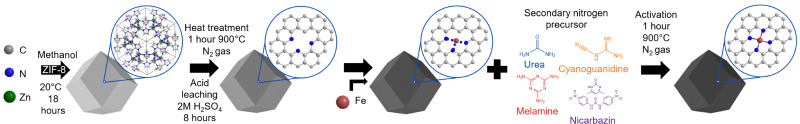
Preparation route of the Fe–N–C catalysts from a ZIF-8 template. Schematic representation of the synthesis procedure: ZIF-8 particles are first heat-treated and acid-leached, followed by iron incorporation *via* transmetalation, and finally thermally activated under an inert atmosphere in the presence of a nitrogen precursor. The crystal structure of ZIF-8 was generated from #COD 7249359^52^ using the VESTA software.^53^

In this work, we seek to understand how secondary nitrogen precursors influence the key performance metrics of Fe–N–C catalysts. Here, we synthesize Fe–N–C catalysts by transmetalation using a ZIF-8 template combined with urea, melamine, cyanoguanidine, or nicarbazin as secondary nitrogen precursors (Fig. 1).^23,51^ We systematically investigate how these precursors influence catalyst morphology, nitrogen coordination, and electrochemical performance. These precursors decompose to release ammonia during pyrolysis, offering a controlled way of enhancing nitrogen incorporation without the risks associated with direct ammonia handling. We first assess the resulting catalyst morphology, particle size, and pore size distribution using scanning electron microscopy and nitrogen physisorption measurements. Second, we quantify the nitrogen content and the coordination environment using X-ray photoelectron spectroscopy. Third, we investigate electrochemical kinetics using a rotating ring disc electrode set-up. Fourth and finally, we assess the performance of the catalysts in a single cell PEMFC under H_2_–air. This study aims to elucidate the effects of nitrogen incorporation and active site formation on ORR performance. Through microscopic, physicochemical, and spectroscopic characterization and electrochemical testing, we establish structure–composition–performance relationships of Fe–N–C catalysts. In doing so, this work not only advances the understanding of Fe–N–C catalyst design but also contributes toward the development of safer, scalable, and more cost-effective pathways for fuel cell catalysts.

## Experimental

### Catalyst preparation

The base catalyst synthesis was carried out according to a method described in ref. 23, with adjustments tailored to this study. 13.14 g of methylimidazole (99%, Merck Life Science N.V.) was dissolved in 200 mL of methanol (HPLC grade, >99.9%, Merck Life Science N.V.), and 11.89 g of zinc nitrate hexahydrate (Zn(NO_3_)_2_·6H_2_O >99%, Merck Life Science N.V.) was dissolved separately in 100 mL of methanol solution. These two solutions were then combined and stirred overnight. The resulting particles were isolated through centrifugation, washed in methanol three times and subsequently pyrolyzed in a tube furnace (Garbolite Gero TF3 12/125/600) at 900 °C for 1 hour under a nitrogen gas flow with a heating rate of 3 K min^−1^ (first thermal step in Fig. 1). The zinc in the pyrolyzed product was removed by leaching through refluxing in 2 M sulphuric acid (H_2_SO_4_ 95–97%, Boom B.V.). Following this, for the transmetalation step, 1 g of leached powder was dispersed in 250 mL of methanol containing 1 g of iron(ii) chloride tetrahydrate (Fe(ii)Cl_2_·4H_2_O >99.0%, Merck Life Science N.V.). This mixture was refluxed overnight with constant stirring. The resulting particles were isolated through centrifugation followed by thorough washing with deionized water (Milli-Q Millipore, 18.2 MΩ cm) and additional overnight leaching in 150 mL of 0.5 M H_2_SO_4_ at room temperature. It was then thoroughly washed *via* centrifugation with water and subsequently dried in a vacuum oven at 70 °C overnight.

### Catalyst activation

For the activation, the washed and leached product was mixed with one of the secondary nitrogen precursors; urea (99.0–100.5%, Thermo Scientific Chemicals), melamine (melamine monomer 98.0+%, TCI Europe N.V.), 1-cyanoguanidine (1-cyanoguanidne for synthesis, Merck Life Science N.V.) and nicarbazin (Merck Life Science N.V.) in a weight ratio of 2 : 1 were thoroughly ground using an agate pestle and mortar. The last step involved heating the mixture in an aluminum oxide crucible to 900 °C for 1 hour under a nitrogen gas flow, with a temperature ramping rate of 3 K min^−1^. After activation, the sample was allowed to cool to room temperature, resulting in the final activated catalyst. Two reference samples were prepared: one without the second heat treatment (referred to as unactivated) and one heat-treated without a secondary nitrogen precursor (referred to as heat-treated).

### Physical characterization

Scanning electron microscopy (SEM) images were taken using a Quanta 200 3DFEG (Thermo Fisher Scientific) at an acceleration voltage of 5 kV. Before imaging, the samples were sputter-coated with platinum (40 mA, 60 s) to increase conductivity for better image quality. The average particle sizes were measured using the ImageJ software. A minimum of 200 particles were analyzed per sample; however, for the nicarbazin-activated catalyst, the number was 150 due to limitations in particle shape, providing a limiting number of particles in the imaging area.

A Micromeritics Tristar II plus instrument was employed to evaluate the specific surface area and pore size distribution, utilizing the Brunauer–Emmett–Teller (BET) theory and Barrett–Joyner–Halenda (BJH) model on the adsorption branch of the isotherm, respectively. For measuring physisorption isotherms, nitrogen grade 5.0 was used. Before the measurements, the samples were degassed at 180 °C for 15–18 hours.

The iron content was evaluated using inductively coupled plasma-optical emission spectrometry (ICP-OES) using a Spectroblue ICP. For the preparation, 25 mg of catalyst was oxidized at 550 °C under air for 5 hours to remove the carbon. The iron-containing residue was dissolved in 5 mL of 50% sulfuric acid in a volumetric glass flask and topped up to 50 mL before being diluted 10 times. The samples were filtered using 5 µm PES filters before the measurement.

Surface functionalities were analyzed using a Thermo Scientific K-alpha X-ray photoelectron spectrometer with a monochromatic Al Kα (1486.6 eV) source (72 W, 400 µm spot size) and a 180° double-focusing hemispherical analyzer with a 128-channel detector. Survey and region scans were recorded at 200 eV and 50 eV pass energies, respectively. The background pressure was 2 × 10^−8^ mbar, which increased to 4 × 10^−7^ mbar during charge compensation with an Ar ion gun. CasaXPS software was used for the deconvolution of narrow scans, applying a 70% Gaussian/30% Lorentzian line shape and a Shirley background.^54^

### Rotating ring disk electrode measurements

Electrochemical analysis for the synthesized catalysts was performed using a rotator (WaveVortex, Pine Instruments) with an E6 ChangeDisk rotating ring disk electrode utilizing a glassy carbon insert with a diameter of 5 mm and a platinum ring. The ring collection efficiency was determined to be *N* = 0.22 according to the methods in ref. 55. The electrolyte used was 0.1 M perchloric acid (HClO_4_, 67–72%, TraceSELECT Ultra, for ultratrace analysis). A platinum sheet counter electrode (1 × 3 cm^2^, Mateck, 99.95%) and a reversible hydrogen reference electrode (Hydroflex, Gaskatel) were utilized. Working electrodes were prepared by polishing with 0.05 µm aluminum polishing liquid (Allied High Tech Products, Inc.) and rinsing with ultrapure water to remove the excess polishing liquid.

The ink was prepared by mixing the electrocatalyst with 2 : 1 water : 2-propanol (HPLC grade, >99.9%, Merck Life Science N.V.) and a Nafion dispersion (NS5 5 wt%, QuinTech) such that the ionomer to carbon ratio (I/C) was 0.125, following the ionomer content for a thin-film electrode from a 20% Pt on Vulcan carbon from ref. 56. The catalyst content was 2 mg mL^−1^ for platinum-based catalysts (38.7 wt% Pt/Vulcan XC72-R, Tanaka Kikinzoku Kogyo) and 4 mg mL^−2^ for PGM-free catalysts. The mixture was dispersed using a Qsonica Q500 probe sonicator to obtain a stable suspension. Thereafter,10 µL of ink was dropped onto the glassy carbon and left to dry in a desiccator with an open beaker of 2-propanol.^57^ The loadings were 0.1 mg cm^−2^ for platinum-based catalysts and 0.2 mg cm^−2^ for PGM-free catalysts. The effect of catalyst loading was studied by varying the loadings to 0.1, 0.2, 0.4, and 0.6 mg cm^−2^. To achieve a loading of 0.1 mg cm^−2^, an ink concentration of 2 mg mL^−1^ was used. For higher loadings, the initial ink was applied in multiple layers, up to three times. The catalyst layers were conditioned by performing cyclic voltammetry (CV) between 0.03 and 1.2 V_RHE_ for 50 cycles at 100 mV s^−1^ for platinum-based catalysts to condition the platinum^58^ and between 0 and 1 V_RHE_ for 10 cycles at 10 mV s^−1^ for PGM-free catalysts to obtain stable ORR performance.^23^ The electric double layer capacitance (EDLC) was evaluated by cycling at 5, 10, 20, 50 and 100 mV s^−1^ in a non-faradaic region. The specific capacitance values were obtained from the slope of current *versus* the scan rate. Additionally, the electrochemically active surface area (ECSA) of platinum was determined by cycling between 0.05 and 1.2 V_RHE_ at 20 mV s^−1^ and integrating the area under the H-desorption peaks, using a specific Coulombic charge for hydrogen adsorption/desorption of 210 µC cm^−2^ (ref. 59 and 60) as a quality control step to confirm the quality of the catalyst film.^56^ The rotation rates used for linear sweep voltammetry were 0, 100, 400, 900, 1600 and 2500 rpm, with a scan rate of 20 mV s^−1^ in both O_2_ and Ar-saturated electrolytes. Finally, the LSVs were 100% iR and background corrected by subtracting the iR corrected measurements in argon from the iR corrected measurements in oxygen.^55,58,61^ The onset potential was determined from the linear extrapolation by taking the intersection of the linear fits to the kinetic region and the steepest portion in the mixed (kinetic and mass transport-limited) region. Half-wave potentials were obtained from fitting the limiting current (mass-transport-limited region) and the linear fit in the kinetic region and then taking half of this current value and extracting the corresponding potential. The electron transfer number was calculated using the Koutecký–Levich equation and literature values of an RDE supplier for the same electrolyte, where the kinematic viscosity was 0.0107 cm^2^ s^−1^, diffusion coefficient was 1.4 × 10^−5^ cm^2^ s^−1^ and oxygen concentration was 1.1 × 10^−6^ mol cm^−3^.^62^

### Gas diffusion electrode preparation and single cell PEMFC testing

Gas diffusion electrodes (GDEs) were prepared by mixing 30 mg of catalyst, 4.5 mg carbon black (Vulcan XC 72-R) to increase conductivity, electrode performance, and reproducibility, 0.15 mL 20 wt% Nafion (water-based dispersion, Quintech), 0.2 mL 1-propanol (HPLC grade, >99.9%, Merck Life Science N.V.) and 0.08 mL of ultrapure water. The mixture was sonicated and drop cast onto a 5 cm^2^ gas diffusion layer with a microporous layer (Freudenberg H15C14) on a hot plate at 90 °C. The GDE was transferred to a vacuum oven when visibly dry and left to dry for 15 hours at 60 °C. The contact between the catalyst layer and the Gore-Select M788.12 membrane was improved by hot pressing with a mask to ensure a compression of 20% at 130 °C for 5 minutes under 5 kN. The anode catalyst layer was ultrasonically sprayed using a Sonotek Flexicoat OP3 system onto the membrane with a loading of 0.1 mg_Pt_ cm^−2^ and sub-gasketed using PEN foil (CMC 61 325 0.025 mm). The membrane-electrode assemblies (MEAs) were conditioned by holding the cell voltage at 0.1 V for 15 minutes to ensure humidification of the membrane. Polarization curves were recorded at 80 °C, 100 RH, 1/5 lpm H_2_/air and 150 kPa abs using a FuelCon Evaluator-C70140 (HORIBA FuelCon GmbH) equipped with a cF5/100 HT (BalticFuelCells GmbH) cell fixture with a 5 cm^2^ seven-channel parallel-serpentine graphite flow field (0.5 mm channel width, 0.8 mm depth, and 0.5 mm land). The cell voltage was held for 5 minutes and steady-state currents were averaged over the final 2 minutes. Electrochemical impedance spectroscopy (EIS) was performed (100 kHz–1 Hz, 10 mV amplitude) at each potential to extract high-frequency resistance (HFR) by interpolating the interception with the real axis. Exhaust water samples were taken after each measurement at open circuit voltage (OCV) and analyzed for fluorine content using a Thermo Fisher Integrion ion 206 chromatography system and a Dionex Ionpac AS11 anion column after calibration curves were made using Thermo Scientific™ Dionex™ Combined Seven Anion Standard I.

## Results and discussion

### Structural and morphological changes after thermal activation

**Fig. 2:**
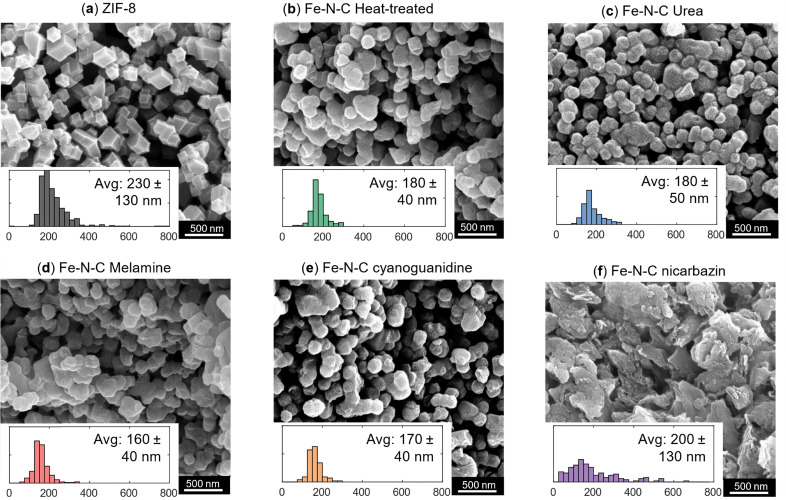
Morphology and particle size distribution of ZIF-8 and Fe–N–C catalysts with SEM. (a) The as-synthesized ZIF-8 and the Fe–N–C catalysts activated in nitrogen using (b) no activating agent, (c) urea, (d) melamine, (e) cyanoguanidine, and (f) nicarbazin, all at a 50 000× magnification. Corresponding histograms show the particle size distribution with size (particle diameter, nm) on the *x*-axis (bin width: 50 nm) and counts (*n*) on the *y*-axis, where each tick is 50 counts.

We first investigate how the morphology and particle size of the activated catalyst (Fig. 2) evolve in relation to the precursor material, ZIF-8. ZIF-8 consists of 2-methylimidazolate as the organic linker, which connects two zinc centers. A characteristic feature of ZIF-8 is its sodalite topology, characterized by six-membered (hexagonal) and four-membered (rhombic) ring windows.^63,64^ Although the molecular framework cannot be resolved using SEM, the particles display well-defined facets and a rhombic dodecahedral morphology (Fig. 2a).

Fig. 2b–f present the morphologies of the catalysts after heat treatment under a nitrogen atmosphere, acid leaching, iron incorporation and finally activation under a nitrogen atmosphere, where Fig. 2b shows the catalyst activated (second heat treatment after iron incorporation) without the use of nitrogen precursors and Fig. 2c–f show the catalyst activated with the use of nitrogen precursors (urea, melamine, cyanoguanidine and nicarbazin). While the particle shape and surface facets become less distinct compared to the pristine ZIF-8, the rhombic dodecahedral morphology is mostly preserved after the heat treatment with the notable exception of the nicarbazin-derived sample. X-ray diffractograms (Fig. S1) reveal that the crystallinity of ZIF-8 is lost during heat-treatment by the disappearance of the ZIF-8 peaks.

During activation, urea, cyanoguanidine, and melamine decompose thermally, releasing gaseous products. In addition, cyanoguanidine and melamine yield carbonaceous residues and graphitic C_3_N_4_.^41–43^ In contrast, nicarbazin undergoes melting prior to decomposition, coating the particles and subsequently breaking down into amorphous carbon and gaseous products.^50,65^ This lack of a predefined decomposition pathway promotes the formation of flake-like carbonaceous residues (Fig. 2f), which entrap the underlying particles.

Across all samples, the average particle size decreases after heat treatment (*e.g.*, 230 ± 130 nm for the ZIF-8 precursor and 180 ± 40 nm after heat-treatment), as shown by the particle size distributions (Fig. 2). This shrinkage is primarily attributed to the pyrolysis of ZIF-8 during the first thermal step, accompanied by zinc removal from the framework, and is consistent for all the catalysts after the activation step with and without nitrogen-containing agents. For the nicarbazin-derived materials, particle size estimation is more challenging due to the flake-like morphology; measurements were performed only on distinguishable particles within the flakes and the flakes themselves, which limits the reliability of the size analysis.

**Fig. 3:**
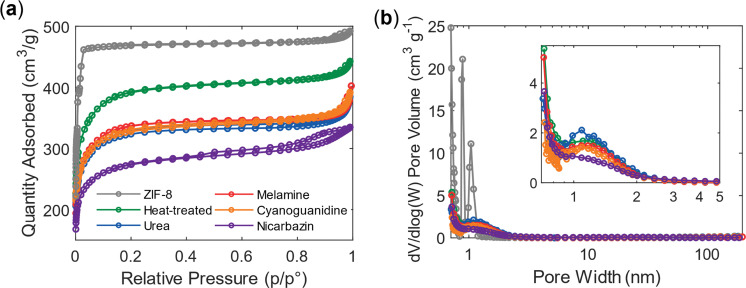
Nitrogen physisorption analysis of the catalysts. (a) Adsorption–desorption isotherms measured at 77 K. (b) Corresponding pore size distributions (PSDs) calculated using the BJH (Barrett–Harkins–Jura) method.^67^

Changes in particle size and structural transformations are also reflected in the corresponding surface area. The isotherm of pristine ZIF-8 (Fig. 3a) exhibits a type I(a) profile, characteristic of microporous solids.^54,66^ At relative pressures approaching *P*/*P*° = 1, a small hysteresis loop appears, which can be attributed to nitrogen physisorption occurring within mesopores arising from monolayer–multilayer adsorption and capillary condensation.^66^ The relatively low magnitude of this hysteresis indicates that mesoporosity represents only a minor fraction of the total pore volume.

For samples activated without an additional agent (*i.e.*, heat-treated), as well as those treated with urea, melamine, or cyanoguanidine, the adsorption profiles shift toward a type I(b) shape with a reduced adsorbed quantity. This suggests that the materials retain comparable, less narrow micro- and mesoporous structures, although their overall surface area decreases. The reduced hysteresis loop further implies a decrease in mesoporosity, which was already limited in the parent ZIF-8 structure and is solely due to the interparticle distance, as ZIF-8 shows no inherent mesoporosity.^64^ In contrast, the nicarbazin-activated sample exhibits a pronounced hysteresis loop, indicating the formation of additional mesopores. These features are most likely generated between flakes or on their surfaces.

BET analysis confirms that thermal activation lowers the total surface area while simultaneously increasing the external surface area (Table 1). This effect is attributed to particle shrinkage (Fig. 2), which increases the surface-to-volume ratio. Despite the reduction, the surface area remains relatively high, and, together with the preserved internal surface area, suggests that the pore network of ZIF-8 is largely maintained. A substantial collapse of the framework would otherwise lead to a drastic loss of porosity and a much lower surface area.

**Table 1:** Calculated BET surface area, micropore volume from the adsorption–desorption isotherms and calculated microporous and external surface area from the *t*-plot method. The error bars represent the standard deviation obtained from two independently synthesized and measured samples

	*S*_BET_ (m^2^ g^−1^)	*V*_micro_ (cm^3^ g^−1^)	*S*_micro_ (m^2^ g^−1^)	*S*_external_ (m^2^ g^−1^)
ZIF-8	1690 ± 60	0.65 ± 0.05	1560 ± 450	80 ± 30
Heat-treated	1430 ± 40	0.435 ± 0.012	1100 ± 28	340 ± 70
Urea	1200 ± 400	0.37 ± 0.13	940 ± 70	290 ± 80
Melamine	1240 ± 20	0.39 ± 0.02	980 ± 280	260 ± 70
Cyano-guanidine	1200 ± 190	0.38 ± 0.06	960 ± 150	280 ± 50
Nicarbazin	700 ± 400	0.19 ± 0.10	470 ± 250	180 ± 120

Because the material is predominantly microporous, pore filling occurs during adsorption rather than capillary evaporation during desorption; therefore, the adsorption branch is used here to provide a representation of the micropore region for BJH analysis. The pore size distribution of pristine ZIF-8 (Fig. 3b) reveals nanometer-sized cages, most likely from the reported interconnected *via* six-membered ring windows.^64^ With the exception of the nicarbazin-activated sample, all samples exhibit a consistent pore size distribution, confirming the preservation of both the framework structure and porosity. For nicarbazin, the observed deviation likely arises from microporous particles being embedded within carbonaceous flakes. A slight shift toward smaller pore sizes was expected for all samples due to shrinkage, although the average pore size became less narrow, but remains within the micropore regime. This retention of microporosity could be advantageous for catalysts in gas-phase reactions;^68^ however, the limited accessibility of micropores to Nafion implies that proton transport could be suboptimal for fuel cell applications.^69^

### Chemical changes after heat treatment

**Fig. 4:**
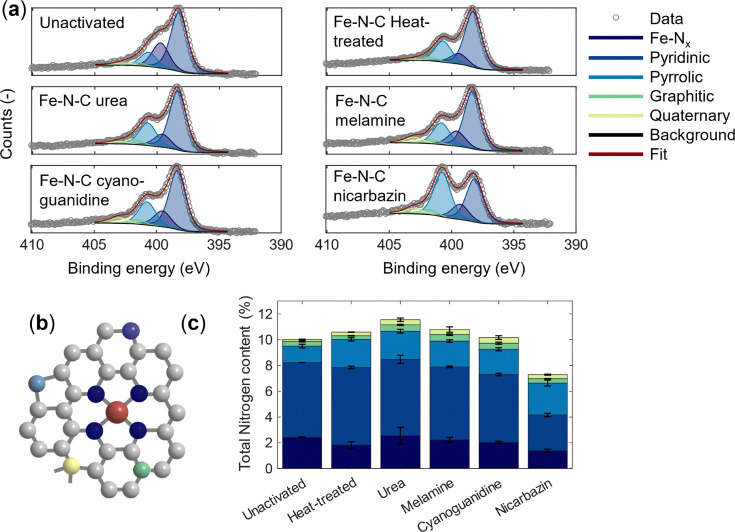
Nitrogen speciation in Fe–N–C catalysts. (a) N 1s spectra of the Fe–N–C catalysts. (b) Schematic representation of coordinated nitrogen species deconvoluted from the N 1s spectra. (c) Absolute amounts of Fe–N, pyridinic, pyrrolic, graphitic, and quaternary groups for the investigated catalyst thermally treated in the presence of the activating agents.^54^ Error bars represent the standard deviation from two independently synthesized and measured samples.

In addition to morphological modifications, the activation process also alters the surface composition and structure (Fig. S2 and Table S1 elemental composition), particularly the nitrogen content and its coordination environment. We evaluate the nitrogen coordination of the catalyst surfaces using XPS (Fig. 4). The total nitrogen content has been linked to catalytic activity,^27^ with higher nitrogen levels generally correlating with improved electrochemical performance. After thermal treatment without a nitrogen precursor, the nitrogen concentration remains essentially unchanged at ∼10 at% compared to the unactivated material (Fig. 4c). By contrast, activation in the presence of secondary nitrogen precursors leads to an increase in total nitrogen content for all agents except nicarbazin.

Nitrogen functionalities are known to play a decisive role in noble-metal-free catalysts for the ORR, as their chemical state strongly influences activity.^70^ In particular, low-energy coordination states have been identified as a key contributor to the most active sites.^27,54^ Across all catalysts, except for the nicarbazin-activated material, the relative proportions of pyrrolic, quaternary, and graphitic nitrogen content remain largely similar (Table S2). However, a significant increase in pyridinic nitrogen in the urea- and melamine-activated samples and an increase in Fe–N_*x*_ species are observed, with the highest concentration in the urea-activated sample, followed by melamine-and cyanoguanidine-activated materials, relative to the control without an activating agent. This trend can be rationalized by differences in the nitrogen release mechanisms of the activating agents.

Urea has been shown to improve pyridinic nitrogen content in the absence of iron in graphene oxide.^28^ Upon decomposition, urea primarily evolves nitrogen in the form of ammonia, which appears to directly promote Fe–N_*x*_ formation as seen in Fig. 4. In contrast, cyanoguanidine and melamine also evolve ammonia but simultaneously generate carbonaceous residues during pyrolysis.^47–49^ In these cases, a fraction of the released nitrogen is likely incorporated into the residues or converted into non-coordinating nitrogen functionalities, rather than being fully available for Fe–N_*x*_ site formation. Consequently, although urea-, melamine-, and cyanoguanidine-derived catalysts exhibit similar total nitrogen contents (∼12 at%), their Fe–N_*x*_ concentrations vary significantly. This indicates that the chemical nature and decomposition pathway of the nitrogen precursor, rather than the total nitrogen content alone, governs the efficiency of Fe–N_*x*_ site formation. This suggests that not all ammonia released during thermal degradation is effectively utilized for Fe–N_*x*_ formation. To assess the effectiveness of nitrogen doping introduced during activation, we perform electrochemical testing.

### Oxygen reduction reaction activity on a rotating disk electrode

**Fig. 5:**
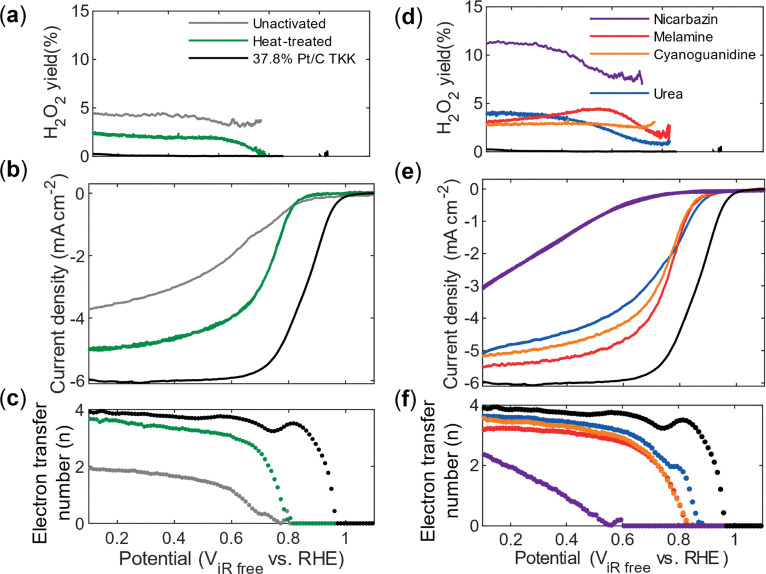
ORR activity and electron transfer of Fe–N–C and Pt/C catalysts. (a) Calculated peroxide yield from ring electrode measurements with the ring potential at 1.2 V. (b) Linear sweep voltammogram (background and iR-corrected) and (c) calculated electron transfer number using the Koutecký–Levich equation for 0.1 mg cm^−2^ platinum on carbon (37.8% Pt/C Vulcan XC-72R, Tanaka) and 0.2 mg cm^−2^ Fe–N–C catalyst before and after thermal treatment, measured in 0.1 M perchloric acid at 1600 rpm (anodic scan, 20 mV s^−1^). (d)–(f) Calculated peroxide yield, linear sweep voltammogram (background and iR-corrected) and calculated electron transfer number using the Koutecký–Levich equation for 0.2 mg cm^−2^ Fe–N–C catalyst thermally treated with activation agents.

The ORR activity of the catalysts was evaluated using rotating ring disk electrode (RRDE) measurements. It has been observed before that the second heat treatment step stabilizes the Fe–N_*x*_ moieties.^71,72^ The effect of the second heat treatment is evident when comparing an unactivated sample (after first thermal treatment, Fig. 1) with a thermally activated sample: a current plateau is not reached for the unactivated sample, an effect attributed to limited accessible active sites, internal mass-transport limitations and/or a high production of hydrogen peroxide consistent with previous reports on Fe–N–C catalysts.^73,74^ The additional heat treatment increases the diffusion limiting current and raises the electron transfer number from ∼2 to nearly ∼4 (Fig. 5a–c). Despite these improvements, a significant performance gap remains between the Fe–N–C catalysts and Pt/C (Fig. 5b).

The use of secondary nitrogen precursors further influences ORR activity, as reflected in the diffusion-limiting current, onset potential, and half-wave potential (Fig. 5d–f). Among the activated catalysts, the urea-activated samples exhibit the highest onset and half-wave potentials (Table 2), which can be explained by a higher content of nitrogen and the Fe–N_*x*_ species, as demonstrated by the XPS results. The catalysts activated with melamine and cyanoguanidine show only modest improvements in onset potential relative to the thermally treated sample without an activating agent. Considering that the XPS results show higher nitrogen and Fe–N_*x*_ content, this modest performance improvement was expected compared to the just heat-treated sample. While melamine slightly enhances the onset and half-wave potentials and diffusion-limiting current, its electron transfer number is lower than that of the cyanoguanidine-derived material. Although the halfway potential is useful to compare platinum-based catalysts with comparable surface features,^75^ the mass activity is more meaningful to compare for PGM-free catalysts.^76^ The mass activities of the Fe–N–C catalysts in Table 2 are much lower (20–350 A g_Fe_^−1^ compared to 1650 A g_Pt_^−1^ for Pt/C at 0.8 V). Here, the mass activity is given by the mass of metal determined through ICP-OES for the Fe–N–C catalysts (Fig. S3 and Table S3). Platinum achieves the highest mass activity; however, among the PGM-free catalysts, an improvement is observed for the urea-activated catalyst (344 A g_metal_^−1^) compared to the heat-treated catalyst (200 A g_metal_^−1^). In contrast, nicarbazin activation results in a decline across all electrochemical performance indicators and is supported by the unfavorable physical performance indicators, *e.g.* smallest surface area and the lowest nitrogen and Fe–N_*x*_ content. This finding highlights that not every nitrogen-containing compound contributes beneficially to active site formation.

**Table 2:** Summary of results from rotating ring disk electrode experiments and ICP-OES for the investigated catalysts measured in 0.1 M HClO_4_ at room temperature (anodic scan, 20 mV s^−1^). The error bars represent the standard deviation obtained from two independently synthesized and measured samples

Catalyst	Specific capacitance (F g^−1^)	ORR onset potential (V)	Halfwave potential (V)	Tafel slope (mV dec^−1^)	Metal (wt% Pt or Fe)	Mass activity 0.8 V (A g_metal_^−1^)
Pt/C	31.4 ± 1.2	0.96 ± 0.007	0.89 ± 0.02	−57 ± 9	37.8	1650
				−180 ± 60		
Fe–N–C-heat-treated	133 ± 8	0.85 ± 0.03	0.753 ± 0.011	−73 ± 3	3.14 ± 0.05	200 ± 60
				−190 ± 20		
Fe–N–C−urea	133 ± 13	0.89 ± 0.010	0.762 ± 0.010	−90 ± 2	3.2 ± 0.4	344 ± 2
				−220 ± 11		
Fe–N–C−melamine	135 ± 6	0.86 ± 0.017	0.75 ± 0.002	−61 ± 17	3.14 ± 0.03	90 ± 50
				−212 ± 5		
Fe–N–C−cyanoguanidine	113 ± 5	0.8548 ± 0.007	0.731 ± 0.011	−99 ± 7	3.216 ± 0.11	157 ± 15
				−238 ± 25		
Fe–N–C−nicarbazin	20 ± 4	0.661 ± 0.002	0.52 ± 0.001		1.7 ± 0.4	26.0 ± 1.2

The electron transfer number was calculated using the Koutecký–Levich equation and the data at 100, 400, 900, 1600 and 2500 rpm in Fig. S4. Platinum shows the highest electron transfer number across the widest potential range, suggesting that the ORR proceeds predominantly *via* the four-electron pathway to water.^19^ Hydrogen peroxide formation is a critical performance metric, as peroxide can trigger degradation of Nafion membranes and ionomers in operating proton exchange membrane fuel cells,^77^ next to decreasing the performance of the cell as it shifts the dominating electron pathway from four to two. The peroxide yield was determined according to ref. 55 with a collection efficiency of *N* = 0.22, see Fig. S5 for the collection efficiency voltammograms. Although linear sweep voltammograms in the diffusion-limited regime appear promising, the Fe–N–C catalysts generate more peroxide than Pt/C, consistent with the higher peroxide yields observed. The ORR can proceed *via* either a two-electron pathway, producing H_2_O_2_ as an intermediate, or a four-electron pathway, producing water directly.^18^ In the associative four-electron pathway, the adsorption energies of the three intermediates (*OOH, *O, and *OH) are not independent but follow scaling relationships, meaning that strengthening or weakening the binding of one intermediate inevitably influences the others. This interdependence makes it challenging to simultaneously achieve optimal adsorption energies for all intermediates and thus limits the attainable catalytic activity. It can be speculated that an increased density of pyridinic nitrogen sites in the activated catalysts may help to locally tune the adsorption environment, improving the balance of intermediate binding energies and thereby favoring the four-electron pathway.^18,34^ From this perspective, the peroxide formation is expected to increase according to the percentage of pyridinic nitrogen in Table S2, from nicarbazin < heat-treated < melamine < cyanoguanidine < urea, which is confirmed through the peroxide yields in Fig. 5a and d.

The ECSA determined from the desorption part of the hydrogen underpotential deposition region for Pt/C is 64 ± 3 m^2^ g_pt_^−1^; this method, however, cannot be applied to the Fe–N–C catalysts. We selected the electrochemical double-layer capacitance (EDLC) as a comparative surface descriptor, since the area-specific capacitance values depend on composition and are not directly comparable across different PGM-free catalysts. The cyclic voltammograms used for EDLC extraction are shown in Fig. S6. Specific capacitance values align with BET surface areas: all Fe–N–C catalysts display high values (∼1000 m_BET_^2^ g^−1^ and ∼130 F g^−1^), except for the nicarbazin-derived sample, which shows significantly lower surface utilization relative to Pt/C (see Table 2).

Tafel slope analysis was performed by plotting the calculated Tafel slopes *versus* the potential (Fig. S7 and S8) and using the constant Tafel slope regions as described in the literature.^78^ In Table 2, the first Tafel slope is reported; slopes for Pt/C in perchloric acid typically fall at −30 and −120 mV dec^−1^,^79^ which is also the case here with a slope of −57 ± 9 mV dec^−1^. A Tafel slope of 59 mV dec^−1^ for platinum is generally attributed to a slow chemical step following a fast electron transfer as the rate-limiting step.^80^ For the Fe–N–C catalysts, the initial Tafel slope falls within the same range (−61 to −99 mV dec^−1^) as Pt/C. The shift to higher Tafel slopes towards 120 arises when the first electron transfer to O_2_ is the rate-determining step.^79^ However, the fact that the Fe–N–C catalysts display similar slopes to Pt/C in this regime suggests that their intrinsic kinetic barrier for O_2_ activation is not significantly higher. At higher overpotentials, however, the Tafel slope increases (−190 to −240 mV dec^−1^ compared to −180 mV dec^−1^ for Pt/C), indicating a shift in the limiting step. This behavior, combined with the observed electron transfer number of ∼3–3.8 and the associated peroxide yield, points toward the partial operation of the 2 × 2 electron pathway. In this pathway, the reduction of adsorbed *OOH species is kinetically hindered, favoring H_2_O_2_ release. Among the Fe–N–C catalysts, the urea- and melamine-activated materials exhibit the most favorable Tafel slopes, attributable to their higher nitrogen content, supporting the conclusion that the local nitrogen environment modulates both activity and selectivity in Fe–N–C catalysts.

**Fig. 6:**
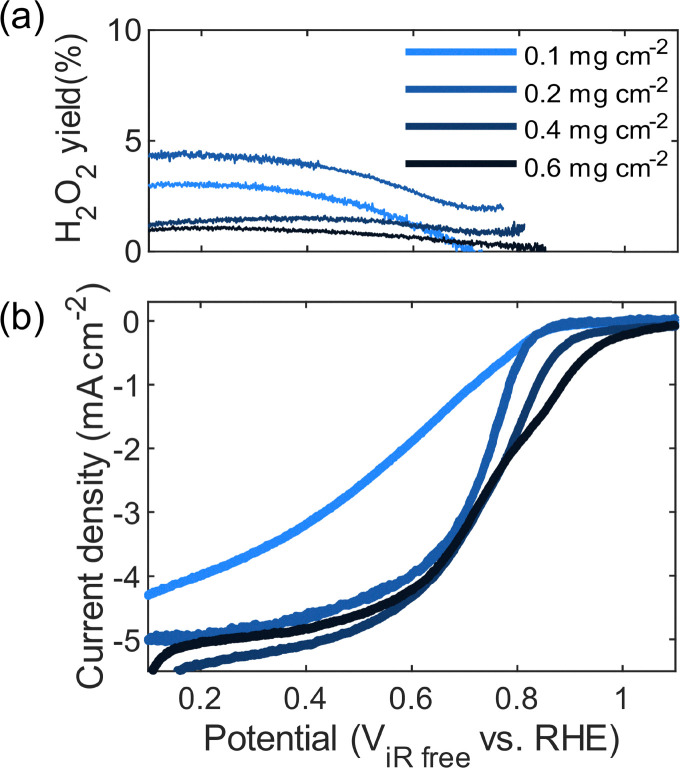
Effect of catalyst loading on the ORR activity of urea-activated Fe–N–C. (a) Calculated peroxide yield from ring electrode measurements with the ring potential at 1.2 V. (b) Linear sweep voltammograms (background and iR-corrected) of 0.1, 0.2, 0.4, and 0.6 mg cm^−2^ Fe–N–C thermally treated with urea in 0.1 M perchloric acid with the ring current at 1.2 V at 1600 rpm (anodic scan, 20 mV s^−1^).

Iron metal can catalyze peroxide formation, representing an additional challenge for Fe–N–C catalysts,^81^ and previous studies have shown that the peroxide formation is related to the catalyst's loading.^82^ To probe the role of catalyst loading, the urea-activated material was tested at four loadings from 0.1 to 0.6 mg cm^−2^ (Fig. 6). We found two notable trends. (i) Increasing the loading from 0.1 to 0.2 mg cm^−2^ increased both the peroxide yield and disk currents at low voltages. (ii) Increasing the loading to 0.4 mg cm^−2^ improved the onset potential and diffusion-limiting current. The initial increase in loading from 0.1 to 0.2 mg cm^−2^ causes a larger number of active sites and thus higher ORR activity, but also higher activity towards peroxide formation. Further increasing the loading from 0.2 to 0.4 mg cm^−2^ raises the onset potential and diffusion-limiting current, while reducing peroxide yield, as peroxide generated within thicker films is more likely to encounter additional active sites and undergo complete reduction to water.^82,83^ A further increase to 0.6 mg cm^−2^ leads to an additional decrease in peroxide yield, indicating suppressed peroxide formation. Since peroxide formation is a critical challenge in PGM-free catalysts, these results highlight increasing catalyst loading as a practical strategy to mitigate peroxide production. Additionally, it also validates that electroanalytical measurements (*e.g.*, RDE and RRDE) are significantly influenced by loading, which challenges precise comparisons across material sets as highlighted in ref. 83.

The onset potential obtained in this work (0.75 V *vs.* RHE at 0.2 mg cm^−2^) is lower than some of the highest values reported in the literature for ZIF-based Fe–N–C catalysts (0.85–0.93 V *vs.* RHE for comparable systems).^20,84^ However, these higher values are often measured at substantially higher catalyst loadings (such as 0.8 mg cm^−2^ (ref. 20)) and under different experimental conditions (including variations in electrolyte composition, catalyst loading, ink formulation, and measurement methodology), which are known to significantly influence the apparent onset potential (as can also be seen in Fig. 6). In contrast, the onset potential reported here compares favorably to other Fe–N–C catalysts evaluated at lower loadings, such as 0.7 V *vs.* RHE (0.4 mg cm^−2^).^85^ Direct comparison across studies remains challenging due to the lack of a standardized testing protocol for Fe–N–C catalysts. A recent literature review^86^ presents a detailed table of half-wave potentials reported for M–N–C catalysts, along with the corresponding catalyst loadings, which illustrates the difficulties in comparing data.

In summary, the intrinsic ORR performance of the synthesized Fe–N–C catalysts was systematically assessed in a three-electrode setup. Onset potential, half-wave potential, limiting current density, and electron transfer number serve as useful indicators of catalytic activity. These measurements clarify how structural and compositional differences among the catalysts translate into electrochemical behavior and provide insight into their prospective performance in fuel cells. Comprehensive stability testing, which falls beyond the scope of the present work, is needed for future studies and requires dedicated protocols, reference materials, and extended operating times. However, as emphasized in previous studies, ORR activity measured in three-electrode configurations does not directly predict performance under practical fuel cell conditions.^87,88^ Therefore, we next assess the Fe–N–C catalysts in single-cell PEMFC hardware to establish their relevance in device operation.

### Performance in a single-cell fuel cell

**Fig. 7:**
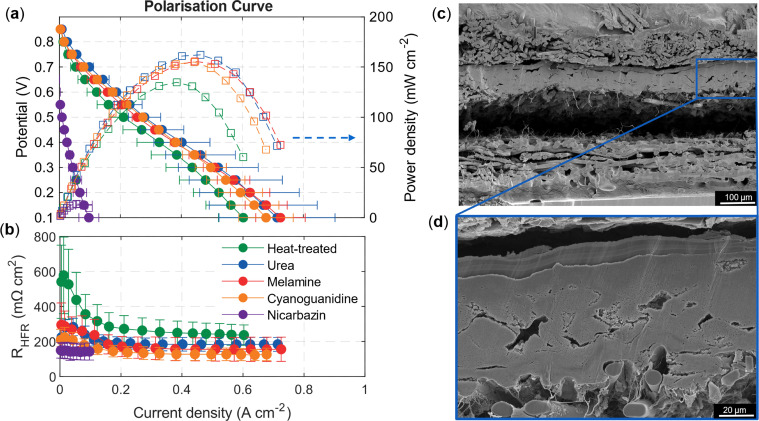
Fuel cell performance of Fe–N–C catalysts. (a) Polarization curves and (b) high-frequency resistance (HFR) measured at 80 °C, 100% RH, and 1/5 L min^−1^ H_2_/air at 1.5 bar_abs_. Cathodes were prepared by drop-casting 4 mg_catalyst_ cm^−2^, while anodes consisted of 0.1 mg_Pt_ cm^−2^ (37.8% Pt/C, Vulcan XC-72, TKK) applied by spray coating. Error bars represent the standard deviation from two independently synthesized and tested samples. (c) and (d) SEM cross-section micrographs of MEA with 4 mg_catalyst_ cm^−2^ Fe–N–C urea drop-cast as the cathode and 0.1 mg_pt_ cm^−2^ (TKK 37.8% Pt–C Vulcan XC-72) as the anode.

Single-cell PEMFC tests were carried out in a 5 cm^2^ (geometric area) fixture with a loading of 4 mg_catalyst_ cm^−2^ (Fig. 7). Several ionomer-to-catalyst ratios and coating methods were investigated, and the results are found in Fig. S9. Catalysts activated with urea, melamine and cyanoguanidine feature comparable performance and slightly outperform the control sample (*i.e.*, the catalyst activated without nitrogen precursors). The improvement is most evident in the kinetic region above 0.6 V (see Fig. S10), where the activated catalysts show higher current densities (*e.g.*, 0.19 A cm^−2^ for the urea-activated catalyst, compared to 0.12 A cm^−2^ for the heat-treated catalyst at 0.6 V). However, the differences are modest and large error bars indicate variability.

The variability is likely linked to the catalyst layer fabrication method. Drop-casting can cause ink penetration into the pores of the gas diffusion layer, interfering with gas transport and product removal. Furthermore, the drop-casting method can result in elevated high-frequency resistance (HFR) values; HFR values for platinum-based catalysts are 1.5 times higher than spray-coated ones, *e.g.* 50–70 compared to ∼30–40 mΩ cm^2^ (Fig. S11), which are hypothesized to be due to poor electronic and ionic conductivity, and contact resistance between the catalyst layer and membrane.^89,90^ When spray-coating Fe–N–C at 4 mg cm^−2^, the resulting layers were bulky and compact, leading to high resistances as well (see Fig. S9d). Although hot pressing was applied for drop-casted MEAs, Fig. 7c and d show local gaps and incomplete contact at the CL/membrane interface resulting in high HFR values of 200–300 mΩ cm^2^. The high loading of the dense Fe–N–C catalyst layers may increase the kinetic performance, but at the cost of mass transport resistance.^91^ Future studies should explore alternative catalyst application techniques to improve layer uniformity and minimize contact resistance for high loadings or thinner Fe–N–C catalyst layers to reduce mass and charge transport resistances.^89–91^

Despite these limitations, we found that the RRDE data (Fig. 4) largely support the fuel cell trends. The control catalyst (heat-treated without nitrogen precursors) showed a slightly lower onset potential, consistent with its lower current density. Among the nitrogen-activated catalysts, the urea-derived sample was expected to be most active based on onset potential, reflected in its higher current density above 0.6 V. However, in the diffusion-limited regime, it did not outperform the melamine- or cyanoguanidine-activated samples. The maximum power density achieved followed the following trend Pt/C > Fe–N–C–urea> Fe–N–C–melamine > Fe–N–C–cyanoguanidine > Fe–N–C-heat-treated > Fe–N–C–nicarbazin with respective values of 679 > 162 > 156 > 155 > 135 > 14 mW cm^−2^. The H_2_/air PEMFC performance obtained in this study is lower than the highest values reported in the literature for PGM-free cathodes (*e.g.*, 562 mW cm^−2^ at 4 mg cm^−2^, 173 kPa, 75 °C, anode 0.1 mg_Pt_ cm^−2^, and 100% RH^92^ or 420 mW cm^−2^ at 4 mg cm^−2^, 100 kPa, 80 °C, and 100% RH^92^ but an anode loading of 2 mg_Pt_ cm^−2^ (ref. 93)), but higher than 50–73 mW cm^−2^ under various conditions.^94–96^ However, direct comparison across studies is complicated by variations in operating conditions and MEA configurations, including temperature, gas stoichiometry, pressure, membrane type, and catalyst loadings. A recent literature review^86^ presents a detailed table of power densities reported for M–N–C catalysts, along with the corresponding operating conditions, which illustrates the difficulties in comparing data. In particular, high-performing reports often employ elevated pressures and higher cathode loadings. In our research, performance is further limited by a relatively high HFR, indicating that ohmic losses and non-optimal MEA properties contribute substantially to the observed results, in addition to the intrinsic activity of the Fe–N–C catalyst.

Consistent with three-electrode measurements, the nicarbazin-activated catalyst shows the poorest performance of the set, as explained by low available surface area, less Fe–N_*x*_ in the structure, and overall sluggish kinetics. As anticipated from RRDE measurements, the Fe–N–C catalysts were outperformed by Pt-based catalysts in terms of current density and onset potential. This highlights that while RDE/RRDE screening provides valuable insight into catalytic activity, MEA performance is additionally governed by the catalyst layer architecture, mass transport, and interfacial resistance, and thus testing under representative fuel cell conditions is needed for reliably comparing materials. To obtain deeper insight into the nature of the Fe–N coordination environment, future efforts should perform X-ray absorption spectroscopy and Mössbauer spectroscopy, but this is beyond the scope of this study.^97^

Peroxide generation, which can accelerate membrane degradation through Fenton-type reactions with iron ions, was also considered here.^77,81^ RRDE results suggested higher peroxide formation for Fe–N–C catalysts compared to Pt/C, in line with their elevated peroxide yields and lower electron transfer numbers. Based on this finding, we evaluate the fluoride ion concentration – as a proxy for membrane and ionomer degradation – by analyzing fuel cell wastewater. The ion chromatography results show marginally higher amounts of fluoride ions in the cell wastewater compared to the Pt/C catalysts (Table 3, with raw data in Fig. S12 and Table S4). Notably, the urea-activated catalyst, which contained the highest nitrogen and Fe–N_*x*_ content, showed the lowest fluoride concentration of 0.123 ± 0.009 mg L^−1^ among all the platinum-free catalysts, suggesting limited membrane degradation under the testing conditions, which is a significant reduction compared to 0.24 ± 0.10 mg L^−1^ for the heat-treated catalyst without the addition of nitrogen precursors.

**Table 3:** Measured amount of fluoride in the wastewater using ion chromatography. The error bars represent the standard deviation obtained from two independently synthesized and measured samples

	Fluoride ions (mg L^−1^)
Heat-treated	0.24 ± 0.10
Urea	0.123 ± 0.009
Melamine	0.19 ± 0.07
Cyanoguanidine	0.18 ± 0.06
Nicarbazin	0.24 ± 0.10
Pt/C (TKK 37.8% Pt Vulcan XC-72)	0.099 ± 0.006

Overall, these findings highlight both consistencies and divergences between RDE and MEA testing. Catalysts activated with nitrogen-rich precursors (urea, melamine, and cyanoguanidine) demonstrated enhanced activity in the kinetic region of PEMFC operation compared to the solely heat-treated sample. However, MEA performance was strongly influenced by the catalyst layer architecture: drop-casting introduced variability through gas transport limitations, poor catalyst layer/membrane contact, and high ohmic resistance. Although RRDE measurements indicated higher levels of peroxide production, ion chromatography showed only marginally higher levels of fluoride concentration in the wastewater across all catalysts, suggesting similar levels of ionomer degradation.

## Conclusions

Fe–N–C catalysts benefit from a nitrogen doping stage to reach improved catalytic activity for non-PGM fuel cell catalyst applications. While ammonia is a common nitrogen source for the high-temperature doping stage, a safer and scalable strategy can be realized by using less volatile nitrogen-containing molecules. We investigated the effect of urea, melamine, cyanoguanidine, and nicarbazin as secondary nitrogen precursors on the physical, chemical, and electrochemical properties of thermally activated Fe–N–C catalysts, as well as their PEMFC performance. The addition of urea, melamine, and cyanoguanidine preserved the structural framework of the catalyst, whereas nicarbazin led to carbonaceous surface coverage and flake-like morphology, resulting in a lower surface area. Among the tested precursors, urea produced the highest nitrogen content and Fe–N_*x*_ species, indicating the most effective doping pathway.

RRDE measurements show that Fe–N–C catalysts activated with urea and melamine exhibited improved onset and half-wave potentials, compared to those synthesized without secondary nitrogen precursors, consistent with their increased nitrogen and Fe–N_*x*_ content. Despite these improvements, a significant performance gap remains between Fe–N–C catalysts and Pt/C across all performance markers, including onset and half-wave potentials, mass activity, Tafel slopes and peroxide formation. Fuel cell tests confirmed the general trends observed in RRDE measurements; secondary nitrogen precursors can enhance the ORR activity of Fe–N–C catalysts. Yet, MEA performance was strongly influenced by the catalyst layer architecture, highlighting the need for optimized fabrication methods. Overall, we found that fuel cell performance is subpar to the state-of-the-art Pt/C catalysts, which motivates further research on iron loading and catalyst layer engineering.

## Author contributions

W. K. contributed to the conceptualization, methodology, validation, formal analysis, investigation, data curation, writing – original draft, writing – review and editing, and visualization. E. B. B. contributed to the supervision and writing – review and editing. M. C. F. contributed to the supervision and writing – review and editing. Finally, A. F. C. contributed to the conceptualization, methodology, funding, resources, writing – original draft, writing – review and editing, project administration, and supervision.

## Conflicts of interest

The authors declare no conflicts of interest.

## Supplementary Material

YA-005-D5YA00357A-s001

## Data Availability

The data presented in this study can be found in the supplementary information (SI) or can be provided by the corresponding author upon reasonable request. Supplementary information is available. See DOI: https://doi.org/10.1039/d5ya00357a.
