# Supplementary material for: Elucidating the influence of secondary nitrogen precursors on the performance of Fe–N–C catalysts for proton exchange membrane fuel cells
Source: Energy Adv. 2026 Feb 20;5(4):571–86. doi: 10.1039/d5ya00357a (PMC12994478; doi:10.1039/d5ya00357a)
Supplement: YA-005-D5YA00357A-s001 [file YA-005-D5YA00357A-s001.pdf]

Supplementary Information (SI) for Energy Advances.
This journal is © The Royal Society of Chemistry 2026

Supplementary Information

Elucidating the influence of secondary nitrogen precursors on the performance of Fe-N-C catalyst for proton exchange membrane fuel cells

Winnie Kong [a], Emre B. Boz [a], Marta Costa Figueiredo[b] and Antoni Forner-Cuenca[a*]

[a]*Electrochemical Materials and Systems, Department of Chemical Engineering and Chemistry, Eindhoven University of Technology, PO Box 513, 5600 MB Eindhoven, Netherlands*

[b]*Electrocatalytic synthesis and electrochemical interfaces, Department of Chemical Engineering and Chemistry, Eindhoven University of Technology, PO Box 513, 5600 MB Eindhoven, Netherlands*

** Corresponding author: a.forner.cuenca@tue.nl*

S1 X-ray diffractogram 2
S2 XPS survey scan 3
S3 ICP-OES calibration 4
S4 RDE data 5
S5 Collection efficiency 6
S6 Specific capacitance 7
S7 Tafel plot 8
S8 Tafel slope 10
S9 Polarization curves PGM-free catalyst using various ionomer ratios and coating methods 11
S10 Polarization curve drop-casted Pt/C 12
S11 Polarization curve Fe-N-C kinetic region 11
S12 IC calibration 13
Tables 14
S1 Elemental composition of the catalysts 14
S2 Nitrogen coordination 14
S3 Iron content from ICP-OES 15
S4 Fluoride concentration in fuel cell water 15

S1 X-ray diffractogram

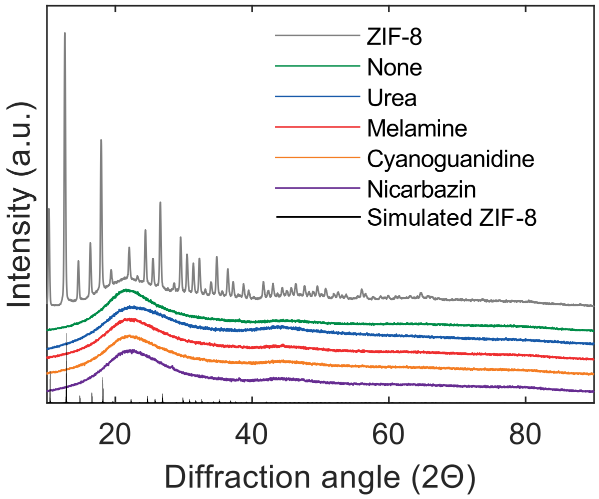

Figure S1: X-ray diffractograms of ZIF-8, its simulated pattern from COD database # 7249359, and the investigated catalysts using a Rigaku MiniFlex 600. The XRD patterns display the characteristic peaks of ZIF-8 and the disappearance of these peaks in the final catalyst.

S2 XPS survey scan

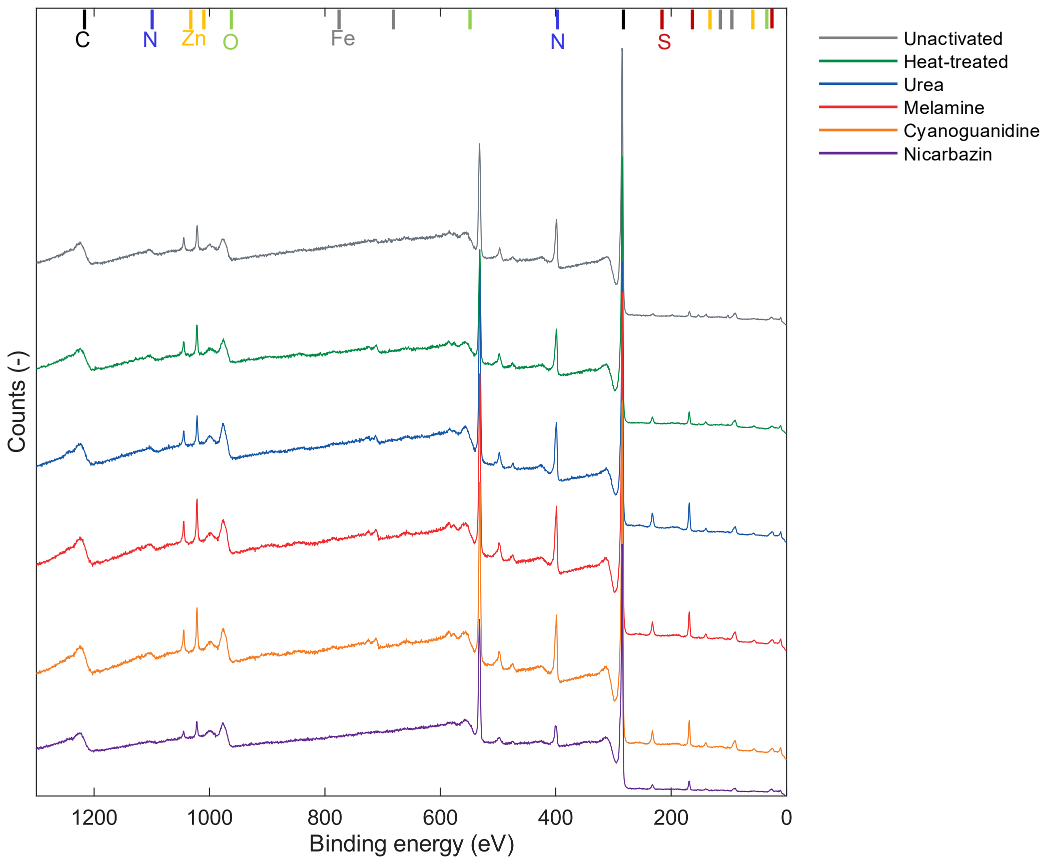

Figure S2: XPS survey spectra of the investigated catalyst thermally treated in the presence of the activating agents and before activation (grey).

S3 ICP-OES calibration

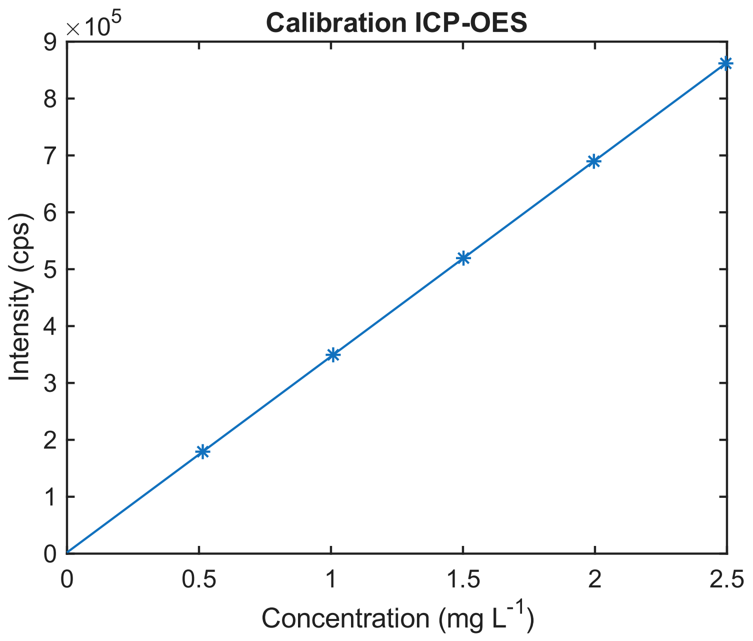

Figure S3: ICP-OES calibration using iron in 0.05% sulphuric acid ($R^2 \approx 0.99998$) in a Spectroblue ICP.

S4 RDE data

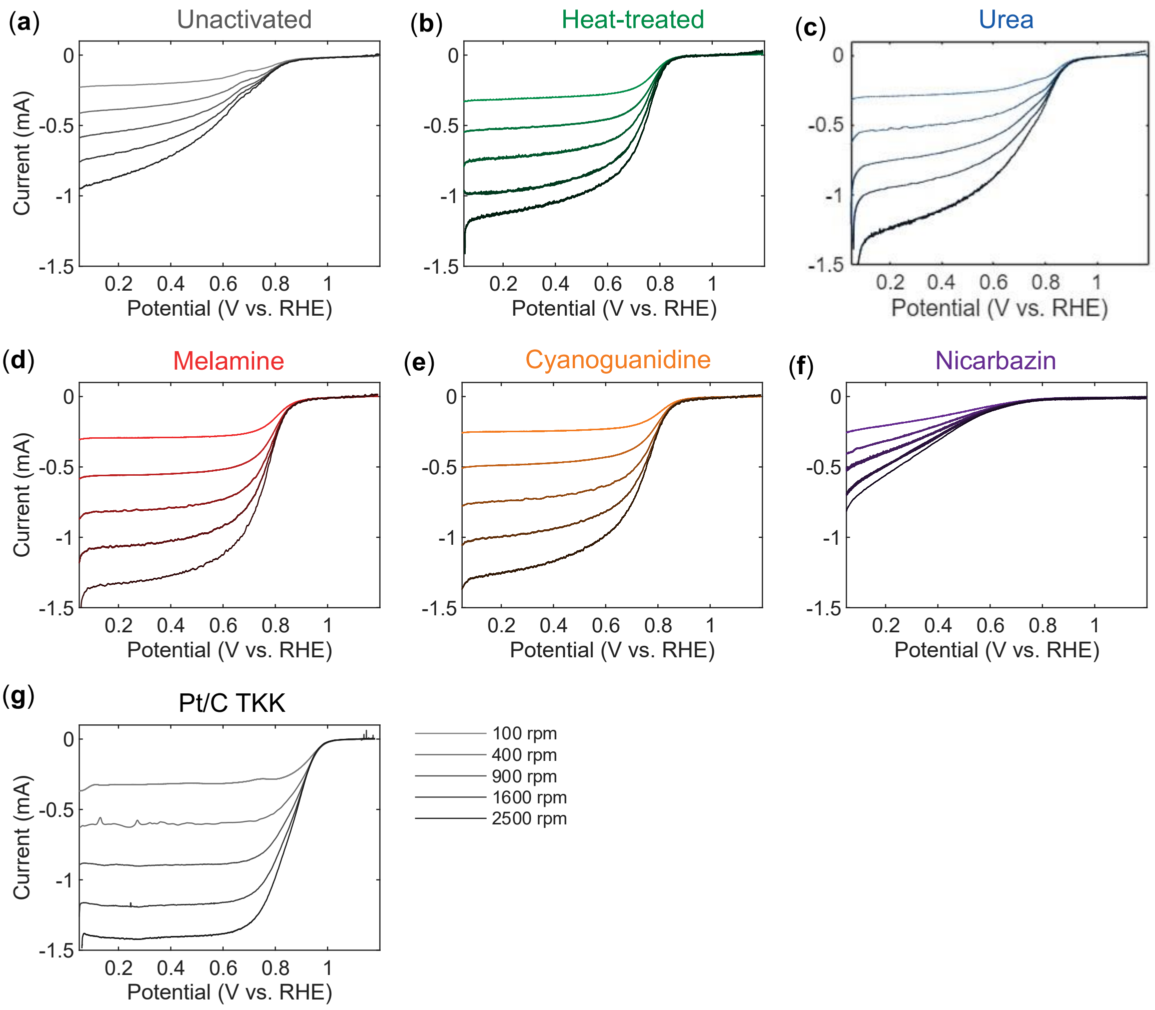

Figure S4: Linear sweep voltammograms (background and iR-corrected) for (**a-f**) 0.2 mg cm^{-2} Fe-N-C catalyst before and after thermal treatment and (g) 0.1 mg cm^{-2} platinum on carbon (37.8% Pt/C Vulcan XC-72R, Tanaka), measured in 0.1 M perchloric acid at 100, 400, 900, 1600 and 2500 rpm (anodic scan, 20 mV s^{-1}).

S5 Collection efficiency

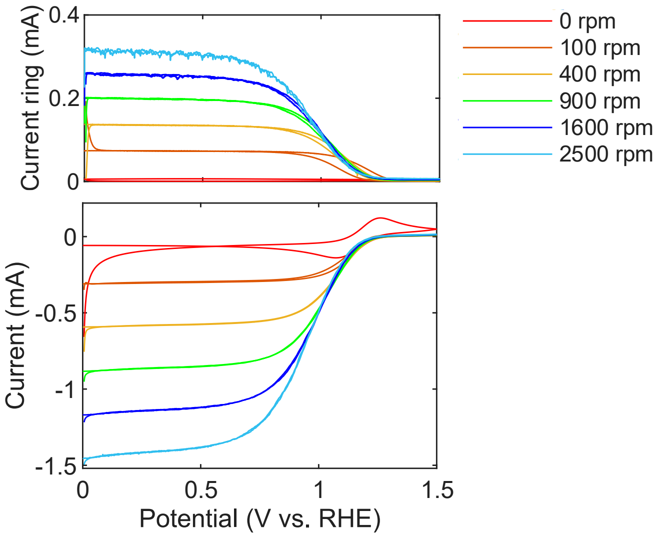

Figure S5: Ring and disk currents at 20 °C for the determination of the collection efficiency on a 0.1 mg cm^{-2} platinum on carbon (19.8% Pt/C Vulcan XC-72R, Tanaka) RRDE in 0.1 M NaOH supporting electrolyte with 10 mM potassium ferricyanide. Positive and negative sweeps at 20 mV s^{-1} and ring potential held at 1.55 V.

S6 Specific capacitance

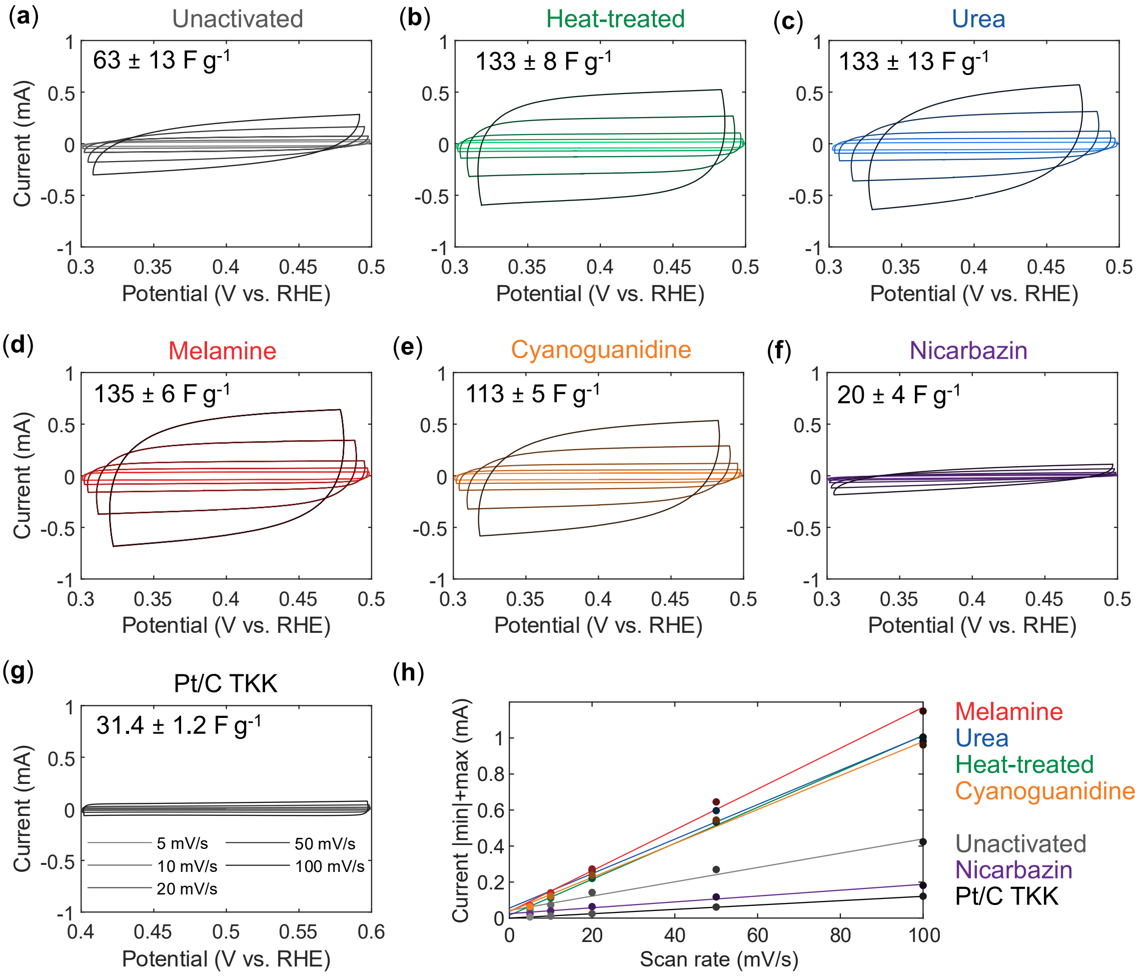

Figure S6: **(a-g)** Cyclic voltammograms of the investigated catalyst in 0.1 M $HClO_4$ using scan rates of 5,10,20,50 and 100 mV s^{-1} in a non-faradaic region. A linear slope is fitted through the obtained current at several scan rates at 0.5 V. The specific capacitance is determined using half of the linear slope in (**h**). The errors correspond to the standard deviation of 2 independent measurements.

S7 Tafel plot

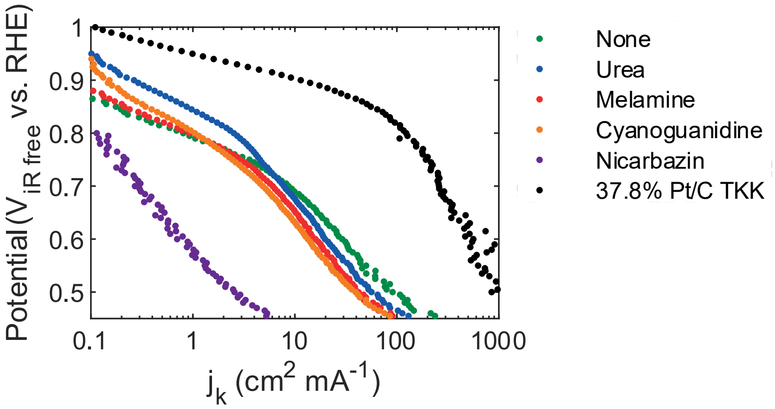

Figure S7: Tafel plots of the investigated Fe-N-C and Pt/C catalysts. Slopes are calculated from linear sweep voltammetry at 1600 rpm with 0.1 mg cm^{-2} platinum on carbon (37.8% Pt/C Vulcan XC-72R, Tanaka) and 0.2 mg cm^{-2} Fe-N-C catalyst before and after thermal treatment, measured in 0.1 M perchloric acid at 1600 rpm (anodic scan, 20 mV s^{-1}).

S8 Tafel slope

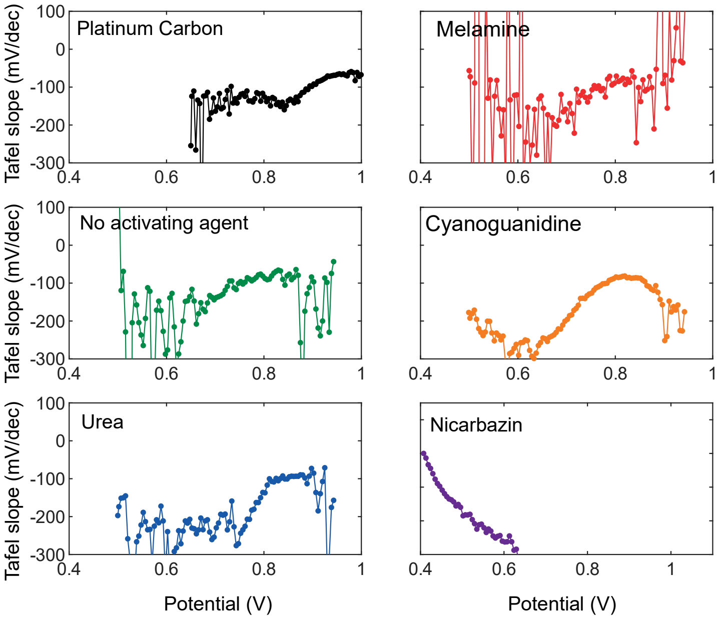

Figure S8: Calculated Tafel slopes for the *Fe-N-C and Pt/C catalysts*. Slopes are calculated from linear sweep voltammetry at 1600 rpm with 0.1 mg cm^{-2} platinum on carbon (37.8% Pt/C Vulcan XC-72R, Tanaka) and 0.2 mg cm^{-2} Fe-N-C catalyst before and after thermal treatment, measured in 0.1 M perchloric acid at 1600 rpm (anodic scan, 20 mV s^{-1}). The numbers in **Table 3** are taken as the stable part of these plots using 2 independent measurements.

S9 Polarization curves PGM-free catalyst using various ionomer ratios and coating methods

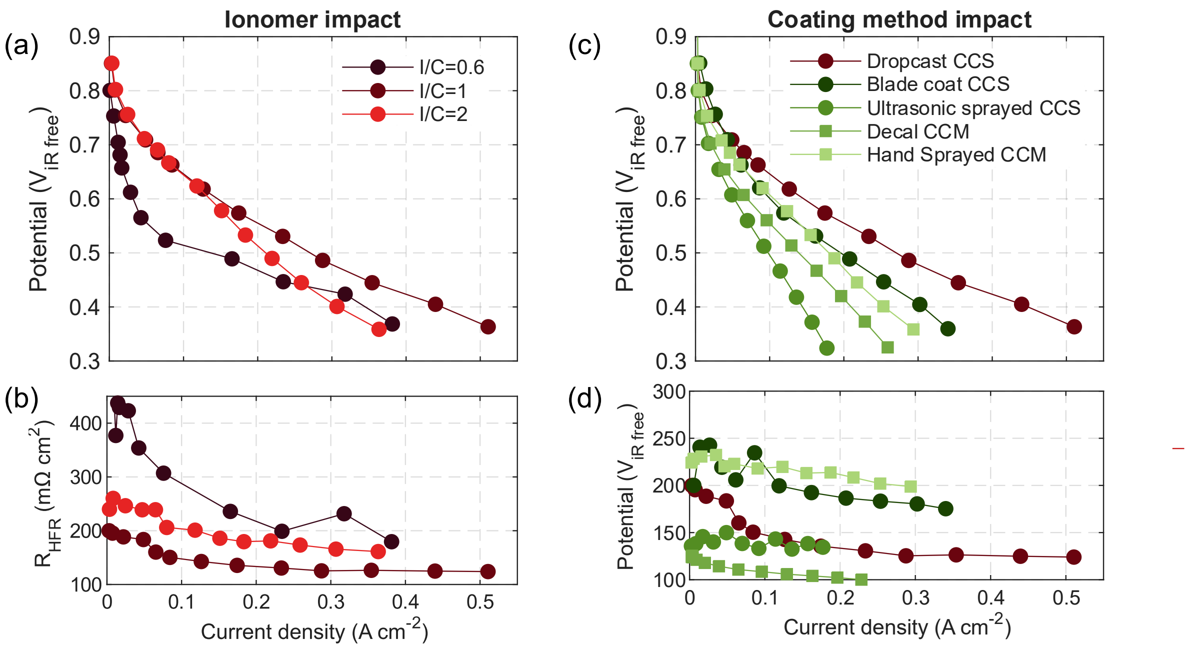

Figure S9: Single cell fuel cell tests with Fe–N-C (urea) as the cathode catalyst in PEMFC. (**a, b**) Drop-casted catalysts with various I/C ratios and (**c, d**) several coating methods (N=1). All loadings are ~4 mg cm^{-2} except decal ~1-2 mg cm^{-2}. Testing conditions: Fuelcon, 5 cm^2, H15C14, 80°C, 100% RH, 0.5/5 lpm H_2/air, 1.5 bar$_{abs}$, 0.1 mg$_{Pt}$ cm^{-2} anode.

S10 Polarization curve Fe-N-C kinetic region

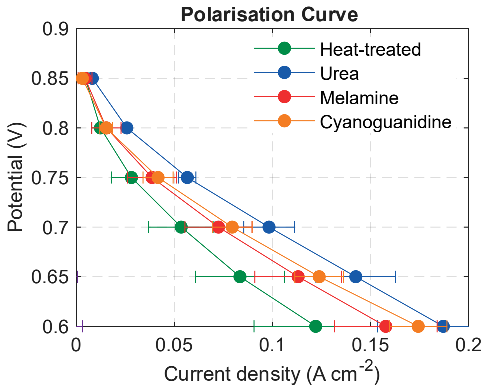

Figure S10. *Fuel cell performance of Fe-N-C catalysts in the kinetic region*. Polarization curves and measured at 80 °C, 100% RH, and 1/5 L min^{-1} H_2/air at 1.5 bar$_{abs}$. Cathodes were prepared by drop-casting 4 mg$_{catalyst}$ cm^{-2}, while anodes consisted of 0.1 mg$_{Pt}$ cm^{-2} (37.8% Pt/C, Vulcan XC-72, TKK) applied by spray coating. Error bars represent the standard deviation from two independently synthesized and tested samples.

S11 Polarization curve drop-casted Pt/C

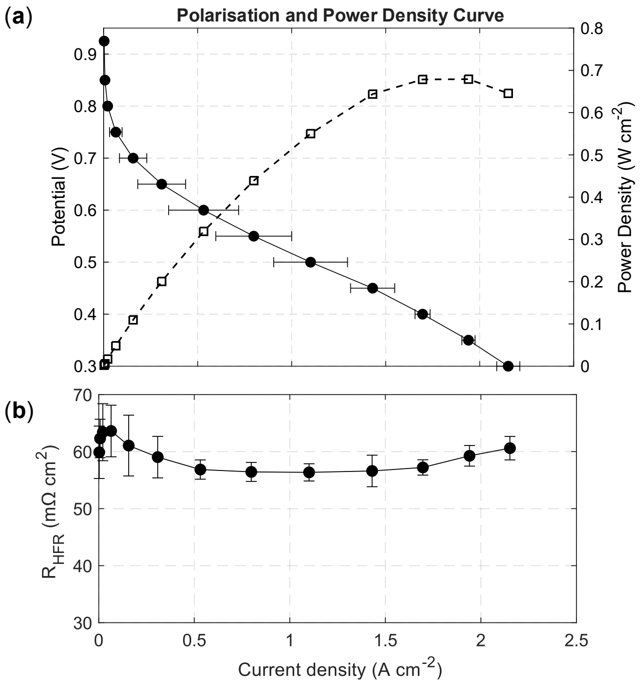

Figure S11: (a) Polarization curve and (**b**) HFR measurements of. 0.4 mg_{pt} cm^{-2} TKK 37.8% Pt-C Vulcan XC-72 drop casted as the cathode and 0.1 mg_{pt} cm^{-2} sprayed as the anode using Freudenberg H15C14, at 80°C, 100% RH, 1/5 lpm H_2/air and 1.5bar_{abs}.

S12 IC calibration

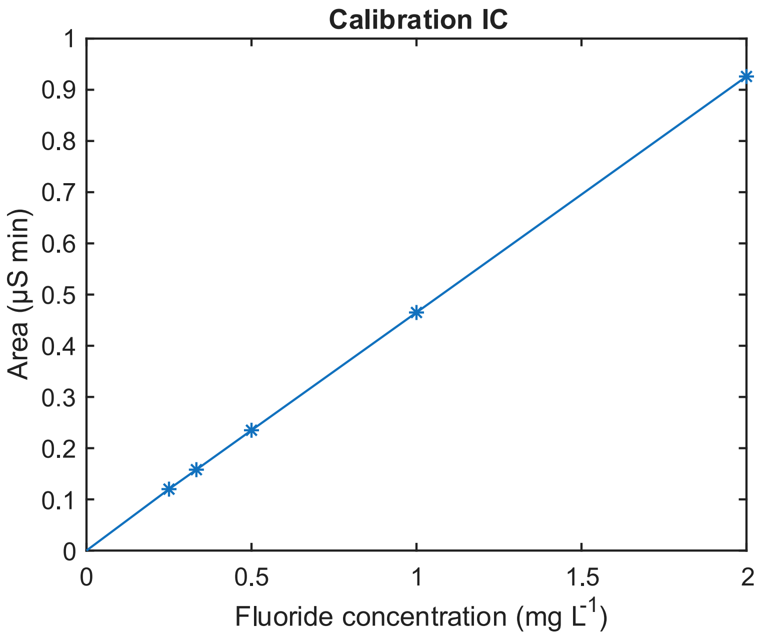

Figure S12: Calibration curve of ion chromatography using Thermo Scientific™ Dionex™ Combined Seven Anion Standard I ($R^2 \approx 0.9999997$).

Tables

S1 Elemental composition of the catalysts

Table S1: The average elemental compositions of the Fe-N-C catalysts from XPS 2 independent measurements. Raw XPS survey scan can be found in **Figure S2**.

Element (at%)	C	N	O	Zn	Fe
**Unactivated**	76 ± 3	10.2 ± 0.7	13.55 ± 3	0.4 ± 0.2	0.08 ± 0.04
**Heat-treated**	79 ± 3	9.1 ± 0.6	9 ± 2	0.9 ± 0.4	0.10 ± 0.05
**Urea**	75 ± 3	11.3 ± 0.7	13 ± 3	0.7 ± 0.3	0.05 ± 0.03
**Melamine**	78 ± 3	10.7 ± 0.7	11 ± 2	0.8 ± 0.3	0.05 ± 0.03
**Cyanoguanidine**	78 ± 3	10.1 ± 0.5	11 ± 2	6 ± 2	0.09 ± 0.04
**Nicarbazin**	81 ± 3	7.3 ± 0.5	11 ± 3	0.3 ± 0.1	N.D.

The error intervals are calculated as the propagation of the largest standard deviation of the series for each element.

S2 Nitrogen coordination

Table S2: The average nitrogen coordination components of the Fe-N-C catalysts (relative %). The average was taken from independently synthesized catalysts per sample.

	Fe-Nx	Pyridinic	Pyrrolic	Graphitic	Quaternary
**Unactivated**	24 ± 5	57 ± 9	13 ± 3	3.6 ± 0.7	2.6 ± 1.1
**Heat-treated**	17 ± 4	57 ± 10	22 ± 4	3.7 ± 0.6	1.4 ± 0.7
**Urea**	23 ± 6	53 ± 10	19 ± 4	2.6 ± 0.4	2.7 ± 1.4
**Melamine**	21 ± 5	53 ± 10	19 ± 4	4.4 ± 0.7	3.5 ± 1.9
**Cyanoguanidine**	20 ± 5	52 ± 10	19 ± 4	4.7 ± 0.8	3.6 ± 1.9
**Nicarbazin**	19 ± 5	38 ± 7	34 ± 7	4.6 ± 0.8	4.3 ± 2.2
**Peak binding energy (eV)**	399.7	398.5	400.7	401.6	402.8
**Literature values (eV)**	399.5-400.5 [1]	398.8 [1]	400.2–400.5 [1]	400.2-401.8 [1]	403 [1]

The error intervals are calculated as the propagation of the largest standard deviation of the series for each element.

[1] K. Artyushkova, Misconceptions in interpretation of nitrogen chemistry from x-ray photoelectron spectra, J. Vac. Sci. Technol. A 38 (2020) 031002. https://doi.org/10.1116/1.5135923

S3 Iron content from ICP-OES

Sample	catalyst (mg)	average (mg L^{-1})	Wt.% Fe
**Heat-treated**	24.5	1.515	3.09%
	25.2	1.607	3.19%
**Urea**	25.4	1.829	3.60%
	25.3	1.462	2.89%
**Melamine**	24.8	1.542	3.11%
	25.9	1.640	3.17%
**Cyanoguanidine**	24.5	1.600	3.26%
	24.6	1.503	3.05%
**Nicarbazin**	25	1.054	2.11%
	24.8	0.638	1.29%

Table S3: Measured iron concentrations of the catalyst through ICP-OES. First column is the amount of catalyst used for the measurement, second column is the average concentration of 3 injections, and third is the calculated iron mass in the catalyst. Calibration curve can be found in **Figure S6**.

S4 Fluoride concentration in fuel cell water

	Sample 1	Sample 2
**Heat-treated**	0.338	0.137
**Urea**	0.134	0.112
**Melamine**	0.266	0.120
**Cyanoguanidine**	0.234	0.117
**Nicarbazin**	0.340	0.145
**Pt/C**	0.093	0.104

Table S4: Estimated fluoride concentrations of fuel cell wastewater (mg L^{-1}). The calibration curve can be found in **Figure S12**. The measured values fall below the lowest calibration standard (0.25 mg L^{-1}). Although the calibration curve exhibits excellent linearity ($R^2 \approx 0.9999997$), concentrations in this range should be considered estimates rather than fully validated values.
